# Seroprevalence of SARS-CoV-2-specific antibodies in the town of Ariano Irpino (Avellino, Campania, Italy): a population-based study

**DOI:** 10.2144/fsoa-2020-0203

**Published:** 2021-01-12

**Authors:** Pellegrino Cerino, Annachiara Coppola, Palmiero Volzone, Antonio Pizzolante, Biancamaria Pierri, Luigi Atripaldi, Massimo Zollo, Mario Capasso, Paolo Antonio Ascierto, Maria Triassi, Gianfranco Brambilla, Alessandro Perrella, Dario Bruzzese, Carlo Buonerba

**Affiliations:** 1 Centro di Referenza Nazionale per l'Analisi e Studio di Correlazione tra Ambiente, Animale e Uomo, Istituto Zooprofilattico Sperimentale del Mezzogiorno, Portici 80055, Italy; 2 Dipartimento di Medicina Sperimentale, Universita’ degli studi della Campania “L. Vanvitelli”, Naples 80138, Italy; 3 Department of Medicine, Surgery & Dentistry (Scuola Medica Salernitana), University of Salerno, Baronissi 84081, Italy; 4 Cotugno Hospital, AORN Ospedali dei Colli, Naples, Italy; 5 CEINGE Biotecnologie Avanzate, Naples, Italy; 6 Unit of Melanoma, Cancer Immunotherapy & Development Therapeutics, Istituto Nazionale Tumori IRCCS Fondazione Pascale, Naples, Italy; 7 Department of Public Health, Federico II University of Naples, Naples, Italy; 8 Istituto Superiore di Sanità, Food Safety, Nutrition, & Veterinary Public Health Department, Rome, Italy; 9 Hospital Health Direction, Infectious Disease Unit, A. Cardarelli Hospital, Naples, Italy; 10 Regional Reference Center for Rare Tumors, Department of Oncology & Hematology, AOU Federico II of Naples, Naples 80131, Italy

**Keywords:** Ariano Irpino, COVID-19, epidemiology, SARS-CoV-2

## Abstract

The Italian municipality of Ariano Irpino (Avellino, Campania, Italy) was locked down by the regional authorities from March until April 2020 after several citizens tested positive for SARS coronavirus 2 (SARS-CoV-2). A serological mass screening campaign targeting the Ariano Irpino population using the Roche Cobas Elecsys anti-SARS-CoV-2 assay was organized by the Zoo-Prophylactic Institute of Southern Italy (Portici, Italy) and conducted in cooperation with the Local Health Unit (Azienda Sanitaria Locale – ASL – Avellino, Avellino, Italy), the Department of Public Health of University Federico II (Naples, Italy) and Department of Health Services of Azienda Ospedaliera dei Colli-Cotugno and Monaldi Hospital (Naples, Italy) in May 2020. A total of 13,218 asymptomatic individuals were reviewed in this analysis. A total of 738 citizens tested positive for anti-SARS-CoV-2 antibodies (398 females, 340 males). The overall prevalence in the sample was 5.6% (95% CI: 5.2–6.0). Among seropositive citizens, 101 cases tested positive on RT-PCR (0.76% of the overall population). Among citizens aged 14–18, 18–65 and >65 years, the seroprevalence was equal to 6.1 (95% CI: 4.1–8.7), 5.6 (95% CI: 5.1–6.1) and 4% (95% CI: 3.3–4.8), respectively. In the pediatric cohort (<14 years old), seroprevalence was 13% (95% CI: 10.2–16.2). A serological-based screening strategy could be a cost-effective public health intervention to tackle the COVID-19 pandemic.

## Lay abstract

A mass screening campaign targeting the Ariano Irpino population in Italy, including a total of 13,218 asymptomatic individuals, showed that 5.6% of the tested individuals had antibodies against the virus responsible for COVID-19 in the blood. Among citizens aged <14, 14–18, 18–65 and >65 years, the proportion of positive individuals was equal to 13, 6.1, 5.6 and 4%, respectively. These asymptomatic individuals, who were identified by a simple blood draw using an inexpensive test (costing only a few euros), could inadvertently spread the infection. A serological-based screening strategy could therefore be a cost-effective public health intervention.

With over 50 million cases and more than 1.2 million deaths reported to the WHO as of November 13, 2020 [1], COVID-19, caused by SARS coronavirus 2 (SARS-CoV-2), represents a global threat that has posed major diagnostic and therapeutic challenges for healthcare professionals [2]. After the first COVID-19 cases were diagnosed in Wuhan (China) in December 2019, the disease quickly spread throughout the world, and COVID-19 was officially classified as a pandemic by WHO in March 2020 [3]. SARS-CoV-2 is a highly contagious virus [4] that can infect the host through small droplets capable of transmitting the infection both when they are breathed in directly or when they land on objects and surfaces [5]. SARS-CoV-2 can exist in aerosols for several hours and on fomites for days [6]. COVID-19 is a multiorgan disease that can affect the lungs, heart, gastrointestinal tract and nervous system [7]. Although overall mortality associated with COVID-19 has been reported to be below 5% in large population-based cohorts [8], it can be as high as 50% in patients admitted to the intensive care unit [9].

In the municipality of Vo', a small town near Padua (Italy) of 3269 inhabitants that was locked down by the regional authorities after several COVID-19 cases were reported and a citizen – the first victim in Italy – died of the disease [10], a large survey based on molecular testing showed a prevalence below 3%, with 42.5% (95% CI: 31.5–54.6%) of confirmed SARS-CoV-2 infections reported in asymptomatic individuals [11]. This result is consistent with data obtained in population cohorts showing asymptomatic/mild disease in approximately 80% of infected individuals and severe disease requiring hospitalization and respiratory support in about 20% of patients [8]. For this reason, epidemiological surveillance can miss most infected individuals, whereas a population-based molecular survey is most likely to identify the majority of asymptomatic individuals but presents practical hurdles due to the cost of RT-PCR molecular testing and processing time. Conversely, seroepidemiological surveys have the advantage of providing population data on past exposure to the virus and may also allow us to reach, at a reasonable cost, large proportions of the population, who may be offered molecular testing in case of seropositivity. Several serological population-based surveys of SARS-CoV-2 have been carried out worldwide, both at a national and municipality level. In the Seroepidemiological Survey of SARS-CoV-2 Virus Infection in Spain, examining >60,000 participants throughout the entire national territory, citizens were assessed for anti-SARS-CoV-2 nucleocapsid IgG antibodies using both a point-of-care rapid antibody test and a chemiluminescent microparticle test [12]. In the SEROCoV-POP study, 2766 inhabitants of Geneva, Switzerland, were assessed for anti-SARS-CoV-2 IgG antibodies using an ELISA-based test [13].

The municipality of Ariano Irpino (Avellino, Campania, Italy), the second largest town of the Avellino province after Avellino itself, covers an area of 186.74 km^2^ and has more than 20,000 inhabitants. The geographical complexity of the municipality territory is reflected in the fragmented distribution of its residential areas, which are often located many kilometers away from each other. The entire town was locked down by the regional authorities from March until April 2020 after several dozen cases were diagnosed. As part of a containment strategy, a serological mass screening campaign using the Roche Cobas Elecsys anti-SARS-CoV-2 chemiluminescence immunoassay (Roche Diagnostics), targeting the entire population of the municipality, was organized by the local health authorities in May 2020. A retrospective analysis of the data collected was conducted to explore seroprevalence differences according to age and sex as well as potential patterns of intrafamilial spread. Data useful for evaluating the potential sanitary impact of a screening strategy based on serological testing of asymptomatic individuals are also provided in this study.

## Methods

### Participants

A population-based serological screening campaign targeting inhabitants of Ariano Irpino (Campania, Italy) – the Ariano Irpino SARS-CoV-2 screening program – was organized by the Zoo-Prophylactic Institute of Southern Italy (Portici, Italy) and conducted in cooperation with the Local Health Authority (Azienda Sanitaria Locale - ASL - Avellino, Avellino, Italy), Department of Public Health of University Federico II (Naples, Italy) and Department of Health Services of Azienda Ospedaliera dei Colli-Cotugno and Monaldi Hospital (Naples, Italy) in May 2020. Screening was offered to all citizens regardless of age but not to institutionalized citizens (e.g., those hospitalized or in prison). Written informed consent to the procedures and collection of sensitive and personal data was obtained from all participants. For citizens aged <18 years, consent was provided by parents or a legal representative.

### Procedures

#### Recruitment

**Figure 1.**
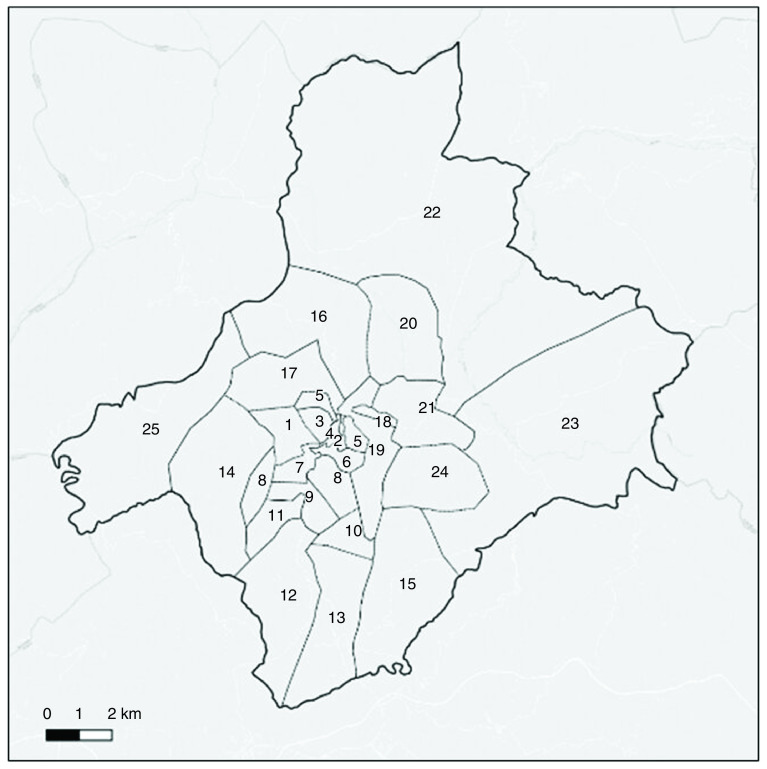
Ariano Irpino territory divided by electoral sections reported in Table 1.

The population of Ariano Irpino consists of 22,246 citizens (10,930 males and 11,316 females); (Italian National Institute of Statistics, updated as of 1 January 2020, provisional data). A meticulous recruitment plan was designed with the aim of reaching >50% of the resident population over 4 consecutive days (days 1, 2, 3 and 4) and implemented in close cooperation with the municipal administration and local nonprofit associations. Electoral lists were provided by the municipality administration and employed to estimate the number of subjects to screen and, consequently, the personnel and supply of consumables required by each site. Buildings used as polling stations during elections were used to carry out the screening operations (Table 1 & Figure 1). All building spaces were inspected and sanitized before use. The population was informed through the standard institutional communication channels of the city council (posters, digital information displays, street-to-street public announcements) as well as through a media campaign conducted via social networks, television and local newspapers. General practitioners also contributed at will.

**Table 1.** List of electoral sections and corresponding polling stations used as testing locations in the municipality of Ariano Irpino.

Electoral section	Polling station
1	Scuola Elementare CALVARIO
2	Scuola Elementare PASTENI
3	Scuola Elementare CALVARIO
4	Scuola Elementare CALVARIO
5	Scuola Elementare PASTENI
6	Scuola Elementare CALVARIO
7	Scuola Elementare CALVARIO
8	Scuola Materna Rione S. PIETRO
9	Scuola Elementare CARDITO
10	Scuola Elementare CARDITO
11	Scuola Elementare CARDITO
12	Centro Servizi S. BARBARA
13	Scuola Elementare TORREAMANDO
14	Scuola Materna S. LIBERATORE
15	Scuola Materna ORNETA
16	Parcheggio PIZZERIA GIORGIA CAMPER
17	Parcheggio PIZZERIA GIORGIA_CAMPER
18	Scuola Elementare RIONE MARTIRI
19	Scuola Elementare RIONE MARTIRI
20	Scuola Elementare CERRETO
21	Scuola Materna TURCO Locali Chiesa
22	Scuola Elementare CAMPOREALE
23	Scuola Elementare TESORO
24	Locali Chiesa VASCAVINO
25	Scuola Elementare PALAZZISI

Citizens were instructed to go to the polling station where they were accustomed to voting. To avoid crowding, two turns during the day (morning and afternoon) were organized on days 1 and 2 by alphabetical order. Those who were unavailable to attend their turn could be tested at a single downtown location on days 3 and 4, whereas those who were confined to their houses (e.g., because of a disability) could request to be tested at home. At each sampling site, trained volunteers made sure that appropriate distancing was maintained, and all participants used the recommended protection devices. Personal and sanitary information, including name, surname, date of birth, symptoms, residence and results of previous tests for SARS-CoV-2, as well as names and surnames of household members was gathered, and a sample of peripheral venous blood was collected by health personnel after obtaining written informed consent.

#### Analytical tests

Antibodies against SARS-CoV-2 were qualitatively assessed in peripheral blood using the anti-SARS-CoV-2 Elecsys E2G 300 assay (Roche Diagnostics). The Elecsys anti-SARS-CoV-2 assay (Roche Diagnostics) is an electrochemiluminescence immunoassay that allows the *in vitro* qualitative detection of antibodies (including IgG) against SARS-CoV-2 in human serum and plasma. This test employs a sandwich reaction that includes both biotinylated and ruthenylated SARS-CoV-2 recombinant nucleocapsid antigens incubated with the sample. The adding of streptavidin-coated microparticles allows the complex to be captured magnetically after binding to the solid phase through a biotin–streptavidin reaction. Electrochemiluminescence emission signals are interpolated to generate test results. Testing requires 12 μl of the sample, and the duration of the procedure is 8 min [14].

For the authors' purposes, the Elecsys anti-SARS-CoV-2 immunoassay (Roche Diagnostics) was performed according to the manufacturer's instructions, and assay results were interpreted as follows: cutoff index <1.0, nonreactive/negative for anti-SARS-CoV-2 antibodies; cutoff index ≥1.0, reactive/positive for anti-SARS-CoV-2 antibodies. Individuals who were positive to the serological test underwent nasopharyngeal swab testing using RT-PCR, which was performed according to WHO guidelines [15] in one of the accredited laboratories of the Coronavirus-Network Laboratories (CORONET-LAB) of the Campania region.

### Study design & statistical analysis

STROBE (Strengthening the Reporting of Observational Studies in Epidemiology) recommendations were followed [16]. This retrospective study was based on a convenience sample. Only asymptomatic individuals and those who had not previously tested positive for SARS-CoV-2 on RT-PCR were included in the study. As others have done, asymptomatic citizens were defined as individuals with no fever and cough and with no more than one symptom among diarrhea, joint pain, vomiting, asthenia, sore throat, muscle pain, headache and loss of taste or smell [11]. Citizens without available data concerning age, sex, residence and results of serological and RT-PCR tests performed were excluded from this study. The primary objective was to analyze differences by age and sex in seroprevalence among asymptomatic citizens participating in the screening campaign. The secondary objectives were to estimate the rate of positive RT-PCR tests among seropositive asymptomatic citizens, to estimate seroprevalence among residents of Ariano Irpino and to explore patterns of household spread. For this last purpose, the authors defined seropositive cases with suspect household transmission as those who had at least one other seropositive household member.

Unweighted prevalence estimates were obtained by dividing the number of positive subjects in each stratum by the sample size of the stratum. The corresponding 95% CI was computed using the binomial exact method. Differences among groups with regard to seroprevalence rates were assessed using chi-square test, and post-hoc analysis was based on residuals of Pearson's chi-square statistics using Holm correction for multiplicity. Percentage of positive RT-PCR tests among seropositive asymptomatic citizens can be interpreted as the positive predictive value of the Elecsys anti-SARS-CoV-2 immunoassay (Roche Diagnostics) in the asymptomatic population during outbreaks. All statistical analyses were conducted using R 3.5.2.

## Results

**Figure 2.**
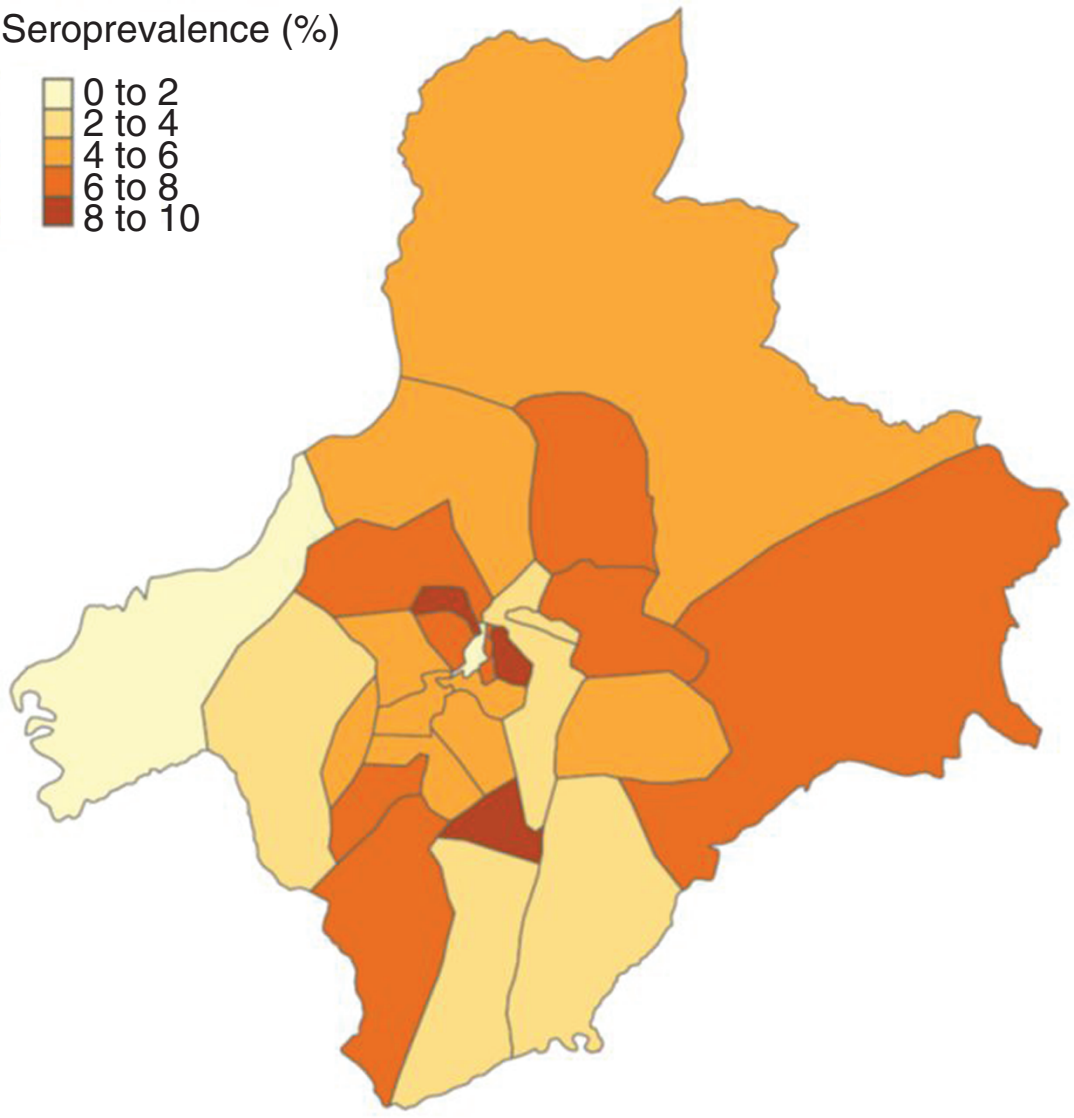
Seroprevalence assessed in Ariano Irpino residents according to electoral sections reported in Table 1.

Of 13,444 individuals screened, a total of 13,218 asymptomatic individuals were recruited during the Ariano Irpino SARS-CoV-2 screening program (May 16–19, 2020), with available data on age, sex, residence and laboratory findings reviewed in this analysis. Median age of the entire cohort was 49 years (interquartile range: 32–62). Approximately 95% of the screened citizens were residents of Ariano Irpino. The age and sex distribution of the entire cohort is reported in Table 2. Considering the total resident population according to updated Italian National Institute of Statistics data, 57.3% of the residents of Ariano Irpino participated in the screening campaign. The pediatric population (<14 years old), including a total of 509 children resident in Ariano Irpino, represented the only age class with an undercoverage of the target population (Table 3). A total of 738 citizens tested positive for anti-SARS-CoV-2 antibodies (398 females, 340 males). The overall prevalence in the sample was 5.6% (95% CI: 5.2–6.0). When considering only residents of Ariano Irpino (n = 12,601), the overall seroprevalence was equal to 5.7% (95% CI: 5.3–6.1). Distribution of seroprevalence among different areas of the municipality territory defined by electoral sections is shown in Figure 2. Significant differences in seroprevalence rates among the age groups were observed (p < 0.001). In the pediatric cohort (<14 years old), which included a total of 516 children, seroprevalence was 13% (95% CI: 10.2–16.2), which was significantly higher than expected based on the entire cohort (p < 0.001). Among citizens in the 14–18, 18–65 and >65 year age groups, the seroprevalence was equal to 6.1 (95% CI: 4.1–8.7), 5.6 (95% CI: 5.1–6.1) and 4% (95% CI: 3.3–4.8), respectively. Similar differences among age groups were reported when the male and female cohorts were considered separately (Table 4). When the authors explored patterns of household transmission with the available data, it was found that 370 (50.1%) positive cases lived in families with a cluster of two or more positive members. The highest involvement in suspected household spread was observed in the younger age groups (household spread was suspected in 68.7, 60.7, 46.9, 51.9% in the <14, 14–18, 18–65 and >65 year age groups, respectively). These differences were statistically significant (Table 5). Serological screening allowed the authors to identify 101 positive RT-PCR cases, representing 13.7% of seropositive cases (95% CI: 11.3–16.4) and 0.76% of the asymptomatic population screened (Table 6). Of the seven positive RT-PCR cases in the <14 year age group, only two were 10 years old or younger. No differences in terms of age, sex and suspected household transmission were found among seropositive individuals who were positive for SARS-CoV-2 on RT-PCR (Table 7).

**Table 2.** Demographic distribution of the entire cohort.

	Overall sample (%)^†^
Overall	13,218 (100)
Age (years)	
0–14	516 (3.9)
15–18	457 (3.5)
19–65	9641 (72.9)
>65	2604 (19.7)
Sex	
Male	6474 (49)
Female	6744 (51)

† Data are reported as absolute frequency (percentage with respect to overall sample/population).

**Table 3.** Demographic distribution of the cohort of residents of Ariano Irpino.

	Participant residents of Ariano Irpino (%)^†^	Ariano Irpino population (ISTAT 2020) (%)^‡^	Participants with respect to corresponding population stratum according to ISTAT (%)
Overall	12,603 (100)	21,995 (100)	57.3
Age (years)			
0–14	509 (4)	2564 (11.7)	19.9
15–18	445 (3.5)	789 (3.6)	56.4
19–65	9082 (72)	13,643 (62)	66.6
>65	2567 (20.3)	4999 (22.7)	51.4
Sex			
Male	6138 (48.7)	10,770 (49)	57
Female	6465 (51.2)	11,225 (51)	57.6

† Data are reported as absolute frequency (percentage with respect to overall cohort).

‡ Data are reported as absolute frequency (percentage with respect to entire population).

ISTAT: Italian National Institute of Statistics.

**Table 4.** Seroprevalence of antibodies against SARS-CoV-2 stratified by age group and sex.

	Overall cohort^†^		Males^‡^		Females^‡^	
	Count	Seropositive (%)	Count	Seropositive (%)	Count	Seropositive (%)
Overall	738	5.6 (5.2–6)	340	5.3 (4.7–5.8)	398	5.9 (5.4–6.5)
Age (years)						
0–14	67	13 (10.2–16.2)	28	11.1 (7.5–15.6)	39	14.8 (10.8–19.7)
15–18	28	6.1 (4.1–8.7)	14	6.4 (3.6–10.5)	14	5.9 (3.2–9.6)
19–65	539	5.6 (5.1–6.1)	248	5.3 (4.7–6)	291	5.9 (5.2–6.6)
>65	104	4 (3.3–4.8)	50	3.8 (2.8–5)	54	4.2 (3.2–5.5)

† Data are reported as absolute frequency (percentage with respect to overall cohort and corresponding age stratum of overall cohort reported in Table 2).

‡ Data are reported as absolute frequency (percentage with respect to overall cohort and corresponding age stratum of overall cohort reported in Table 2 divided by sex).

SARS-CoV-2: SARS coronavirus 2.

**Table 5.** Patterns of household spread of SARS-CoV-2, stratified by age group, according to serology findings.

Household spread	Overall cohort (%)^†^	0–14 (years) (%)^†^	14–18 (years) (%)^†^	18–65 (years) (%)^†^	>65 (years) (%)^†^
No	368 (49.9)	21 (31.3)	11 (39.3)	286 (53.1)	50 (48.1)
Yes	370 (50.1)	46 (68.7)	17 (60.7)	253 (46.9)	54 (51.9)

† Data are reported as absolute frequency (percentage with respect to overall seropositive cohort and corresponding age stratum of overall cohort of seropositive cases reported in Table 4).

SARS-CoV-2: SARS coronavirus 2.

**Table 6.** Rates of RT-PCR SARS-CoV-2-positive cases, stratified by age group and sex, in the subgroup of seropositive citizens.

	Overall cohort^†^		Males^‡^		Females^‡^	
	Count	Positive (%)	Count	Positive (%)	Count	Positive (%)
Overall	101	13.7 (11.3–16.4)	42	12.4 (9–16.3)	59	14.8 (11.5–18.7)
Age (years)						
0–14	7	10.4 (4.3–20.3)	2	7.1 (0.9–23.5)	5	12.8 (4.3–27.4)
15–18	0	0 (0–12.3)	0	0 (0–23.2)	0	0 (0–23.2)
19–65	78	14.5 (11.6–17.7)	31	12.5 (8.7–17.3)	47	16.2 (12.1–20.9)
>65	16	15.4 (9.1–23.8)	9	18 (8.6–31.4)	7	13 (5.4–24.9)

† Data are reported as absolute frequency (percentage with respect to overall cohort and corresponding age stratum of overall seropositive cohort reported in Table 4).

‡ Data are reported as absolute frequency (percentage with respect to overall seropositive cohort and corresponding age stratum of overall seropositive cohort reported in Table 4 divided by sex).

RT-PCR: Real-time PCR; SARS-CoV-2: SARS coronavirus 2.

**Table 7.** Patterns of household transmission of SARS-CoV-2, stratified by age group, according to RT-PCR results.

Household transmission	Overall cohort (%)^†^	0–14 (years) (%)^†^	14–18 (years) (%)^†^	18–65 (years) (%)^†^	>65 (years) (%)^†^
No	44 (43.6)	1 (14.3)	0 (0)	35 (44.9)	8 (50)
Yes	57 (56.4)	6 (85.7)	0 (0)	43 (55.1)	8 (50)

† Data are reported as absolute frequency (percentage with respect to overall seropositive cohort and corresponding age stratum of overall cohort of seropositive cases reported in Table 6).

RT-PCR: Real-time PCR; SARS-CoV-2: SARS coronavirus 2.

## Discussion

The serological population-based screening campaign conducted in May 2020, targeting citizens dwelling in the municipality of Ariano Irpino in the Avellino province, showed an overall seroprevalence of 5.6% (95% CI: 5.2–6.0). The authors' results are consistent with those obtained in population cohorts recruited during the pandemic at a national, regional or municipality level. In a nationwide population-based survey conducted in Spain, a total of 61,075 participants were tested for SARS-CoV-2 by a point-of-care antibody test and, optionally, by a chemiluminescent microparticle immunoassay. Seroprevalence was 5.0% (95% CI: 4.7–5.4) by the point-of-care test and 4.6% (95% CI: 4.3–5.0) by immunoassay, with a lower seroprevalence in children younger than 10 years of age. Importantly, only approximately a third of seropositive participants were asymptomatic [12]. In another seroprevalence survey conducted in the population of Geneva, Switzerland, a total of 2766 participants from 1339 households were tested weekly for 5 weeks for anti-SARS-CoV-2 IgG antibodies using a commercially available ELISA test. Seroprevalence was 4.8% (95% CI: 2.4–8.0; n = 341) in the first week, 8.5% (95% CI: 25.9–11.4; n = 469) in the second week, 10.9% (95% CI: 27.9–14.4; n = 577) in the third week, 6.6% (95% CI: 24.3–9.4; n = 604) in the fourth week and 10.8% (95% CI: 28.2–13.9; n = 775) in the fifth week. Importantly, participants in the age range of 5–9 years (relative risk: 0.32; 95% CI: 0.11–0.63) and those >65 years (relative risk: 0.50; 95% CI: 0.28–0.78) showed a significantly lower risk of being seropositive than those in the age range of 20–49 years [13]. One study has shown that children are less likely to develop symptomatic infection, with the probability of having clinically meaningful COVID-19 dropping from approximately 70% in older adults to approximately 20% in children [17]. Furthermore, in a study of 1775 residual samples collected in 1076 children requesting medical attention at Seattle Children's Hospital, a seroprevalence of ≈1% was reported [18]. In a Dutch cohort of 3207 citizens aged 2–90 years, seroprevalance estimates were 4.9% in the 18–39 year age group compared with 1.7% in children 2–17 years of age [19]. In the current study, the authors found a significantly higher than expected seroprevalence in the 0–14 year age group (13 compared with 5.6% of the overall population), which may reflect the non-random selection of participants but may also be the result of household transmission. In fact, 68.7% of seropositive cases in the 0–14 year age group presented at least one seropositive family member versus 60.7, 46.9 and 51.9% of participants, respectively, in the 14–18, 18–65 and >65-year age groups, with a statistically significant difference. Also, the authors found only two children aged 10 or younger positive on RT-PCR, which is consistent with the findings obtained in the Vo' study, where none of the 217 and 157 children in this age group tested during the first and second surveys, respectively, were positive on RT-PCR [11].

Overall, the authors' screening campaign allowed the identification of 101 asymptomatic individuals who were subsequently found positive on RT-PCR and may have contributed to the spread of the infection. Of note, after Ariano Irpino was locked down by the regional authorities from March 15 to April 22, 2020, and the screening campaign was completed in May 2020, the town was declared COVID free in July 2020 [20]. The authors are unable to assess the exact contribution provided by identification of asymptomatic carriers of SARS-CoV-2, who represented approximately 0.7% of the asymptomatic population assessed in the screening campaign. Of note, the authors' results appear consistent with those obtained in the Vo' experience [11], where 29 asymptomatic individuals, representing approximately 1% of the 2812 participants undergoing molecular testing at the first survey, were found positive on RT-PCR.

Our retrospective analysis suffers from multiple limitations, including lack of a sample size computation, non-random sample selection and lack of a longitudinal serological assessment, which others have performed [12,13]. Also, we are unable to estimate how many citizens who may have been positive on RT-PCR at the time of screening were missed by serological assessment. Nevertheless, it must be acknowledged that we recruited more than half of the resident population in only 4 days and identified 101 asymptomatic individuals, representing ∼0.7% of the assessed population, who may have spread the infection and may have possibly never been diagnosed. Also, only 5% of citizens had to undergo molecular testing, which shows that this strategy may be cost-effective during outbreaks, even compared with using RT-PCR on pooled samples [21,22], with the additional advantage of providing seroprevalence data. Seropositive patients may also be tested using more sensitive techniques, such as droplet digital PCR [23]. Finally, the authors' findings show a higher seroprevalence in children, which represents a novelty. This result may simply reflect a distortion of the sample due to the non-random selection of participants and the undercoverage of this age group; however, a true higher circulation of the virus in children of Ariano Irpino cannot be excluded.

## Conclusion

The screening containment strategy applied to the municipality of Ariano Irpino was feasible and potentially cost-effective. It also provided baseline data for follow-up studies that can establish temporal trends in seroprevalence at a municipality level that may reflect trends at a regional or national level. Finally, this approach may represent an attractive alternative to RT-PCR testing on pooled samples for mass screening of SARS-CoV-2 in asymptomatic citizens.

## Future perspective

This large population-based study shows how, with proper organization, thousands of individuals can be enrolled in a screening campaign in only a few days. Despite the challenges posed by the pandemic, >50% of Ariano Irpino's population was enrolled in 4 days. The experience reported is to be intended as a public health intervention, which may be successfully applied in the future should other biological or nonbiological threats to the majority of the population emerge. For instance, it could be employed in biomonitoring studies to investigate the levels of biological contamination at an individual level and assess the effects on human health. Additional studies are required to assess the economic sustainability of such interventions.

**Summary points**

- COVID-19 is a global threat. Asymptomatic individuals can be responsible for spreading the disease.
- Mass screening using serological tests can assess seroprevalence and allow early identification of asymptomatic individuals.
- Among 13,218 asymptomatic individuals recruited during the Ariano Irpino SARS coronavirus 2 (SARS-CoV-2) Screening Program (16–19 May 2020), the overall prevalence in the sample was 5.6% (95% CI: 5.2–6.0). Serological screening allowed the authors to identify 101 cases positive on real-time PCR, representing 13.7% of seropositive cases (95% CI: 11.3–16.4) and 0.76% of the asymptomatic population screened.
- In the pediatric cohort (<14 years old), including a total of 516 children, seroprevalence was 13% (95% CI: 10.2–16.2), which was significantly higher than expected based on the entire cohort (p < 0.001). Among citizens in the 14–18, 18–65 and >65-year age groups, the seroprevalence was equal to 6.1 (95% CI: 4.1–8.7), 5.6 (95% CI: 5.1–6.1) and 4% (95% CI: 3.3–4.8), respectively.
- The screening containment strategy applied to the municipality of Ariano Irpino was feasible and potentially cost-effective. It also provided baseline data for follow-up studies that can establish temporal trends in seroprevalence at a municipality level that may reflect trends at a regional or national level. Finally, this approach may represent an attractive alternative to real-time PCR testing on pooled samples for mass screening of SARS-CoV-2 in asymptomatic citizens.
